# Probing microdomain Ca^2+^ activity and synaptic transmission with a node-based tripartite synapse model

**DOI:** 10.3389/fnetp.2023.1111306

**Published:** 2023-02-10

**Authors:** Langzhou Liu, Huayi Gao, Jinyu Li, Shangbin Chen

**Affiliations:** ^1^ Britton Chance Center for Biomedical Photonics, Wuhan National Laboratory for Optoelectronics-Huazhong University of Science and Technology, Wuhan, China; ^2^ MoE Key Laboratory for Biomedical Photonics, School of Engineering Sciences, Huazhong University of Science and Technology, Wuhan, China

**Keywords:** astrocyte, microdomain Ca2^+^ activity, tripartite synapse, computational model, fine processes

## Abstract

Astrocytic fine processes are the most minor structures of astrocytes but host much of the Ca^2+^ activity. These localized Ca^2+^ signals spatially restricted to microdomains are crucial for information processing and synaptic transmission. However, the mechanistic link between astrocytic nanoscale processes and microdomain Ca^2+^ activity remains hazily understood because of the technical difficulties in accessing this structurally unresolved region. In this study, we used computational models to disentangle the intricate relations of morphology and local Ca^2+^ dynamics involved in astrocytic fine processes. We aimed to answer: 1) how nano-morphology affects local Ca^2+^ activity and synaptic transmission, 2) and how fine processes affect Ca^2+^ activity of large process they connect. To address these issues, we undertook the following two computational modeling: 1) we integrated the *in vivo* astrocyte morphological data from a recent study performed with super-resolution microscopy that discriminates sub-compartments of various shapes, referred to as nodes and shafts to a classic IP_3_R-mediated Ca^2+^ signaling framework describing the intracellular Ca^2+^ dynamics, 2) we proposed a node-based tripartite synapse model linking with astrocytic morphology to predict the effect of structural deficits of astrocytes on synaptic transmission. Extensive simulations provided us with several biological insights: 1) the width of nodes and shafts could strongly influence the spatiotemporal variability of Ca^2+^ signals properties but what indeed determined the Ca^2+^ activity was the width ratio between nodes and shafts, 2) the connectivity of nodes to larger processes markedly shaped the Ca^2+^ signal of the parent process rather than nodes morphology itself, 3) the morphological changes of astrocytic part might potentially induce the abnormality of synaptic transmission by affecting the level of glutamate at tripartite synapses. Taken together, this comprehensive model which integrated theoretical computation and *in vivo* morphological data highlights the role of the nanomorphology of astrocytes in signal transmission and its possible mechanisms related to pathological conditions.

## 1 Introduction

Over the past few decades, astrocytes have raised a great concern (Volterra and Meldolesi, 2005; Sofroniew and Vinters, 2010; Molofsky and Deneen, 2015; Vainchtein and Molofsky, 2020). Increased evidence show that astrocytes actively participate in the brain functions (Hertz and Chen, 2016; Verkhratsky and Nedergaard, 2018; Deitmer et al., 2019) and importantly, they are hallmark of various brain diseases (Burda and Sofroniew, 2014; Verkhratsky et al., 2014; Sofroniew, 2015; Pekny et al., 2016). Astrocytes are electrically non-excitable cells that respond to stimulation with elevations of Ca^2+^ concentration. These Ca^2+^ signals appear as “global” and/or “focal” responses within the astrocyte complex (Guerra-Gomes et al., 2017). Three-dimensional Ca^2+^ imaging shows that 80% of total Ca^2+^ activity happens in fine processes which occupy 75% of cell volume and form the so-called spongiform domain (Bindocci et al., 2017). Understanding the Ca^2+^ signals involved is crucial to reveal the special role of astrocytes in health and diseases (Escartin et al., 2021).

Recently, great interests and technical advances have significantly promoted the topic. By taking advantage of high-resolution two-photon microscopy, Di Castro et al. found an intense local Ca^2+^ activity in the processes of mature astrocytes, called “focal” and “expanded” events due to the short duration and high frequency (Di Castro et al., 2011). Moreover, the local Ca^2+^ transients are closely associated with synaptic function. Bindocci et al. provided the first comprehensive 3D map of Ca^2+^ activity in an individual astrocyte, thereby demonstrating its complexity, heterogeneity, and locality, notably at the astrocyte-synapse interface, where activity is small, fast, and frequent (Bindocci et al., 2017). Stobart et al. identified, for the first time, fast astrocyte Ca^2+^ microdomains in fine processes and endfeet by using novel combinations of genetically encoded Ca^2+^ indicators (Stobart et al., 2018). They provided new insight into the timing of activity in the fine structures of astrocyte and neuron, suggesting that astrocyte signaling is fast enough to play a role in synaptic modulation. Arizono et al. performed 3D-STED microscopic imaging of the spongiform domain of astrocytes and observed a reticular meshwork of nodes and shafts that often formed loop-like structures (Arizono et al., 2020). The work also shows that nodes not only host highly localized spontaneous Ca^2+^ transients but also are likely functional components of tripartite synapses. Ding et al. investigated the Ca^2+^ transient changes in the subcellular domain of astrocytes during brain aging (Ding et al., 2022). The results suggest that aging-induced changes of Ca^2+^ transient types are heterogeneous within astrocytic subcellular domains. Despite these advances, established optical techniques typically break down at this nanoscale due to the diffraction limit, making difficult to understand the mechanistic link between their morphology and Ca^2+^ signals.

Computational modeling is an effective way to understand complex systems with a much more systematic and controlled way than what could be done experimentally. The same hold true for studying cellular signalling in astrocytes. In this vein, Cresswell-Clay et al. proposed a minimal compartmental model for astrocytes that can qualitatively reproduce essential hierarchical features of spatiotemporal Ca^2+^ dynamics in astrocytes (Cresswell-Clay et al., 2018). Savtchenko et al. systematically incorporated multi-scale, 3D astroglial architecture into a realistic multi-compartmental cell model (Savtchenko et al., 2018). It is detailed but also much computationally demanding. Gordleeva et al. introduced a spatially extended astrocyte model to study the effects of interplay of Ca^2+^ signals in processes and in Soma mediating correlations between local signals and the cell-level response of the astrocyte (Gordleeva et al., 2019). Denizot et al. used a stochastic spatially explicit individual-based strategy and proposed an IP_3_R-mediated Ca^2+^ signaling model for dynamics in fine processes (Denizot et al., 2019). Subsequently, they modelled node-shaft geometries of the gliapil by designing an isolated fine process containing five identical nodes and four identical shafts based on their super-resolution study (Denizot et al., 2022). Verisokin et al. flattened astrocyte images used as spatial templates to reduce the 3D of realistic astrocytes to 2D allowing for smaller computational costs than Savtchenko et al. (2018), Verisokin et al. (2021).

As there are only limited spatial models without full consideration of the morphological parameters on Ca^2+^ activity the integration of experimental data to computational approaches is required Here, to clarify how nano-morphology of astrocytes controls Ca^2+^ activity, we gave a more complete picture by proposing a structurally similar model of realistic astrocytic fine processes using published data from live tissue (Arizono et al., 2020). The model consisted of initial sites of Ca^2+^ microdomains, nodes, connecting shafts, and a section of large process. The variable size of nodes and shafts could cause the diffusion changes of whole dynamics and finally powerfully affected the Ca^2+^ activity involved. Simulations allowed for the explanations of complicated relations between astrocytic morphology and excitability, such as the effect of nano-morphology of fine processes on local Ca^2+^ activity, interactions between fine and larger processes and how pathological morphological deficits of astrocytes affect glutamate-mediated tripartite synapses.

To recap, our study aims to shed light on the effect of the nano-morphology of astrocytic fine processes on local Ca^2+^ microdomain activity and synaptic transmission. We posit this integrated approach could help understand certain mechanisms such as plasticity of astrocyte dynamical processes and pathological morphological changes in brain disorders like Alzheimer’s disease (AD) and inspire new insights into therapies for unsolved neurological diseases (Zhang et al., 2022).

## 2 Materials and methods

Astrocytes display a complex morphology characterized by distinct compartments: the Soma, primary processes, and numerous fine processes which form the astrocytic spongiform domain resembling a reticular meshwork of dividing and merging astrocytic processes. Most of those compose tripartite synapses with their neuron partners where they sense neuronal activity and process this information as a Ca^2+^ signal (Semyanov et al., 2020). Super-resolution imaging shows the anatomical units of those fine processes: nodes and shafts (Arizono et al., 2020). These nodes and shafts constitute a network featuring closely spaced bulbous node-like enlargements that frequently form branch points from which several thinner connecting shaft-like processes emerge. Nodes are simultaneously locations of initial sites of Ca^2+^ signals and the participant of tripartite synapses while shafts may not be capable of generating Ca^2+^ events spontaneously (Arizono et al., 2020).

Considering these, we constructed two submodels, a node-shaft model and a tripartite synapse model. The former investigated how the morphology of fine processes affects Ca^2+^ activity at the astrocyte scale. The node-shaft model consisted of spherical structures, nodes, connected to each other or the parent process with cylindrical structures, shafts. We then extended the original model by adding a neuronal component, hoping to further study the effect of morphological changes of fine processes on neuronal activity in the tripartite synaptic structure. The tripartite synapse model was composed of one of nodes in the former model, a presynaptic bouton and a postsynaptic spine. Nodes possess endoplasmic reticulum (ER) so that Ca^2+^ signals can occur when IP_3_Rs on the ER membrane are at open state. Shafts contain no organelles but IP_3_ and Ca^2+^ can diffuse through them. In the model, the width of nodes was set to greater than or equal to the width of shafts. In summary, these two parts together form the node-based tripartite synapse model.

### 2.1 Ca^2+^ dynamics in nodes

**FIGURE 1:**
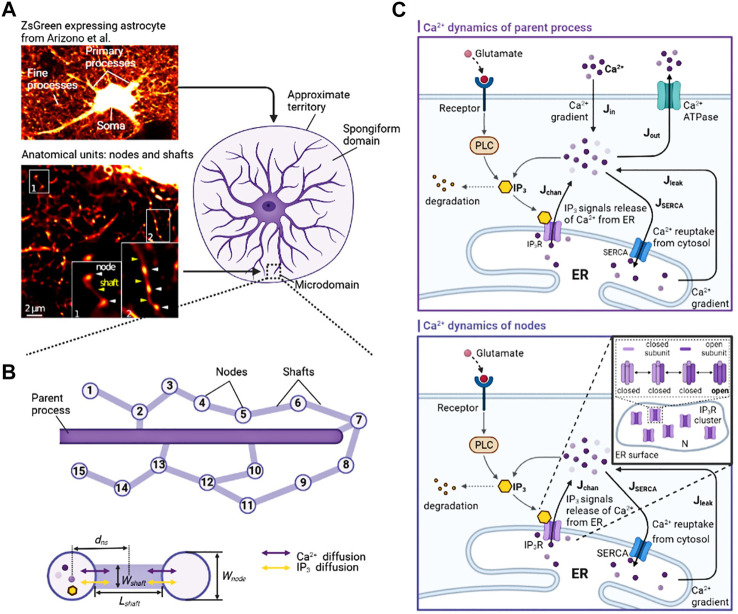
Biophysical models and kinetic schemes used for simulating Ca^2+^ dynamics in node and parent process **(A)** Confocal overview image of astrocytes expressing ZsGreen from Arizono et al. (2020) reveals a complex three-dimensional topology and representative STED image from Denizot et al. (2022) shows the anatomical units of fine processes: nodes and shafts **(B)** The designed similar node-shaft model consists of 15 nodes and the parent process. Nodes have ER and are sites of initiation of Ca^2+^ signals while shafts do not have any organelles. $W_{node}$ is the width of nodes, $W_{shaft}$ is the width of shafts, $L_{shaft}$ is the length of shafts, $d_{ns}$ is the distance between the centers of nodes and shafts **(C)** Biochemical processes included in the different structure of the model. In parent process, Ca^2+^ can entry/exit the cytosol from/to the extracellular space or the ER. The kinetic is based on Ullah et al. (2006) While in the node, Ca^2+^ only has pure intracellular source due to the stochastic opening and closing process of IP3R. We directly simulated this stochastic dynamics by a two-state Markov process, adapted from Li-Rinzel (Shuai and Jung, 2002). [Figure 1A adapted from (Arizono et al., 2020) and (Denizot et al., 2022)].

Ca^2+^ microdomains can have a pure intracellular or extracellular Ca^2+^ source (Lia et al., 2021). As shown in the Ca^2+^ dynamics of nodes in Figure 1C, the IP_3_-dependent Ca^2+^ signaling mediated by channels or pumps on the ER membrane is considered:

$$\frac{d{\left[{Ca}^{2+}\right]}_{i}^{n}}{dt}=J_{chan}^{n}+J_{leak}^{n}-J_{SERCA}^{n}+J_{Cadiff}^{n},$$ (1)

where ${\left[{Ca}^{2+}\right]}_{i}^{n}$ is the intracellular Ca^2+^ concentration and n is the representation of nodes. $J_{chan}^{n}$ represents Ca^2+^ release from ER to cytosol, $J_{leak}^{n}$ is the Ca^2+^ gradient across the ER membrane, and $J_{SERCA}^{n}$ is the Ca^2+^ efflux from the intracellular space back to the ER. $J_{Cadiff}^{n}$ represents the Ca^2+^ diffusion flux.

When IP_3_ binds to IP_3_Rs, Ca^2+^ exits from the open IP_3_Rs and triggers Ca^2+^ signals. Accounting for small volumes of fine processes and the stochasticity of Ca^2+^ signals involved, the kinetics of IP_3_Rs is described by a two-state Markov process (opening state and closing state) derived from the Li-Rinzel model (Li and Rinzel, 1994), also called Markov-stochastic Li-Rinzel model by Shuai and Jung (2002). Specifically, there are several IP_3_Rs on the ER membrane, the open fraction of IP_3_Rs can be directly expressed as the ratio of the number of open IP_3_R channels $N_{h-open}$ to the total number of IP_3_R channels N :

$$J_{chan}^{n}=v_{1}m_{\infty }^{3}n_{\infty }^{3}\frac{N_{h-open}}{N}\left({\left[{Ca}^{2+}\right]}_{ER}^{n}-{\left[{Ca}^{2+}\right]}_{i}^{n}\right),$$ (2)

$$m_{\infty }=\frac{{\left[{IP}_{3}\right]}_{n}}{{\left[{IP}_{3}\right]}_{n}+d_{1}},$$ (3)

$$n_{\infty }=\frac{{\left[{Ca}^{2+}\right]}_{i}^{n}}{{\left[{Ca}^{2+}\right]}_{i}^{n}+d_{5}},$$ (4)

where ${\left[{Ca}^{2+}\right]}_{i}^{n}$ denotes the localized Ca^2+^ concentration released from a cluster of channels, ${\left[{Ca}^{2+}\right]}_{ER}^{n}$ is the Ca^2+^ concentration in the ER with ${\left[{Ca}^{2+}\right]}_{ER}^{n}=\left(c_{0}-{\left[{Ca}^{2+}\right]}_{i}^{n}\right)/c_{1}$ with free Ca^2+^ concentration $c_{0}=2.0\;\mu M$ and $c_{1}$ is the volume ratio between ER and cytosol. $m_{\infty }$ , $n_{\infty }$ , are the gating variables.

In detail, an IP_3_R can exist in four different states, and the kinetic scheme describing the behavior of this channel is given by (Shuai and Jung, 2002):

$$n_{0}\;\underset{\beta_{h}}{\overset{3\alpha_{h}}{⇄}}\;n_{1}\;\underset{{2\beta }_{h}}{\overset{2\alpha_{h}}{⇄}}\;n_{2}\;\underset{{3\beta }_{h}}{\overset{\alpha_{h}}{⇄}}\;n_{3},$$ (5)

where $n_{i}$ is the number of the channels with i open gates and hence $n_{3}$ represents the open state of the IP_3_R channel. $\alpha_{h}$ and $\beta_{h}$ are opening and closing rates respectively. For example, if the channel is at state $n_{1}$ at time t , then the probability that it becomes $n_{2}$ at time t+Δt is $1-\exp\left(-2\alpha_{h}∙\Delta t\right)$ , or it becomes $n_{0}$ at time t+Δt is $1-\exp\left(-\beta_{h}∙\Delta t\right)$ . Only if all three h gates in an IP_3_R channel are open at time t , the channel is h -open. The total population of open IP_3_Rs will be updated for every time step Δt .

The expressions for $J_{leak}^{n}$ and $J_{SERCA}^{n}$ are given by (Shuai and Jung, 2002):

$$J_{leak}^{n}=v_{2}\left({\left[{Ca}^{2+}\right]}_{ER}^{n}-{\left[{Ca}^{2+}\right]}_{i}^{n}\right),$$ (6)

$$J_{SERCA}^{n}=v_{3}\frac{{\left({\left[{Ca}^{2+}\right]}_{i}^{n}\right)}^{2}}{{\left({\left[{Ca}^{2+}\right]}_{i}^{n}\right)}^{2}+k_{3}^{2}},$$ (7)

In Li-Rinzel model [IP_3_] is typically treated as a parameter. Actually, in astrocytes, IP_3_ can be synthesized by both the Ca^2+^-dependent activity of PLCδ and glutamate-dependent activity of PLCβ (De Pitta et al., 2009). Therefore, in our model, ${\left[{IP}_{3}\right]}_{n}$ is associated with the two sources, the kinetics of IP_3_ production and degradation are described as follows (Ullah et al., 2006):

$$\frac{d{\left[{IP}_{3}\right]}_{n}}{dt}=J_{PLC\beta }^{n}+J_{PLC\delta }^{n}-\frac{1}{\tau_{ip3}}\left({\left[{IP}_{3}\right]}_{n}-{\left[{IP}_{3}\right]}_{n}^{*}\right)+J_{{IP}_{3}diff}^{n},$$ (8)

with

$$J_{PLC\beta }^{n}=v_{\beta }\frac{g^{0.3}}{g^{0.3}+k_{g}^{0.3}}.$$ (9)

$$J_{PLC\delta }^{n}=v_{4}\frac{{\left[{Ca}^{2+}\right]}_{i}^{n}+\left(1-\alpha \right)k_{4}}{{\left[{Ca}^{2+}\right]}_{i}^{n}+k_{4}},$$ (10)

where $J_{PLC\beta }^{n}$ and $J_{PLC\delta }^{n}$ represent the PLCβ- and PLCδ-dependent IP_3_ production, respectively. The third part describes IP_3_ degradation and enforces a steady state ${\left[{IP}_{3}\right]}_{n}^{*}$ . $J_{{IP}_{3}diff}^{n}$ represents the IP_3_ diffusion flux from other compartments. g denotes the amount of glutamate applied to the model, we kept g = 1 μM in our simulations.

### 2.2 Ca^2+^ dynamics in parent process

We used modified stochastic Ullah’s model to describe the Ca^2+^ dynamics of the parent process (Ullah et al., 2006). This part of the modeling is graphically shown in the Ca^2+^ dynamics of parent process in Figure 1C. The whole Ca^2+^ dynamics are composed of six components including the Ca^2+^ release from ER to cytosol $J_{chan}$ , the leakage flux from the ER to cytosol $J_{leak}$ , the uptake from cytoplasm to ER with the ATP-dependent pump $J_{SERCA}$ , the plasma membrane influx $J_{in}$ , Ca^2+^ extrusion $J_{out}$ and Ca^2+^ diffusion from the fine processes $J_{Cadiff}$ :

$$\frac{d{\left[{Ca}^{2+}\right]}_{i}}{dt}=J_{chan}+J_{leak}-J_{SERCA}+J_{in}+J_{out}+J_{Cadiff},$$ (11)

$$\frac{dh}{dt}=\alpha_{h}\left(1-h\right)-\beta_{h}h+\sigma_{h}dw_{h},$$ (12)

$$\frac{d{\left[{Ca}^{2+}\right]}_{ER}}{dt}=\left(J_{chan}+J_{leak}-J_{SERCA}\right)/c_{1},$$ (13)

$$\frac{d\left[{IP}_{3}\right]}{dt}=J_{PLC\beta }+J_{PLC\delta }-\frac{1}{\tau_{ip3}}\left(\left[{IP}_{3}\right]-{\left[{IP}_{3}\right]}^{*}\right)+J_{{IP}_{3}diff},$$ (14)

with

$$J_{chan}=v_{1}{m_{\infty }^{3}n_{\infty }^{3}h}^{3}\left({\left[{Ca}^{2+}\right]}_{ER}-{\left[{Ca}^{2+}\right]}_{i}\right),$$ (15)

$$J_{leak}=v_{2}\left({\left[{Ca}^{2+}\right]}_{ER}-{\left[{Ca}^{2+}\right]}_{i}\right),$$ (16)

$$J_{SERCA}=v_{3}\frac{{\left[{Ca}^{2+}\right]}_{i}^{2}}{{\left[{Ca}^{2+}\right]}_{i}^{2}+k_{3}^{2}}.$$ (17)

$$J_{in}=v_{5}.$$ (18)

$$J_{out}=k_{1}{\left[{Ca}^{2+}\right]}_{i}.$$ (19)

$$J_{PLC\beta }=v_{\beta }\frac{g^{0.3}}{g^{0.3}+k_{g}^{0.3}},$$ (20)

$$J_{PLC\delta }=v_{4}\frac{{\left[{Ca}^{2+}\right]}_{i}+\left(1-\alpha \right)k_{4}}{{\left[{Ca}^{2+}\right]}_{i}+k_{4}},$$ (21)

where $\sigma_{h}dw_{h}$ is a Wiener process we added to induce the channel noise accounting for the stochastic nature of Ca^2+^ signals (Riera et al., 2011).

### 2.3 Intracellular diffusion of Ca^2+^ and IP_3_

As shown in Figure 1B, the whole microdomain dynamics is formed by the intracellular diffusion of Ca^2+^ ( $J_{Cadiff}^{n}$ ; $J_{Cadiff}$ ) and IP_3_ ( $J_{{IP}_{3}diff}^{n}$ ; $J_{{IP}_{3}diff}$ ) through compartments accounted by the following fluxes:

$$J_{Cadiff}=D_{Ca}\left({\left[{Ca}^{2+}\right]}_{i,1}+{\left[{Ca}^{2+}\right]}_{i,2}+\ldots +{\left[{Ca}^{2+}\right]}_{i,n}-n{\left[{Ca}^{2+}\right]}_{i}\right),$$ (22)

$$J_{{IP}_{3}diff}=D_{{IP}_{3}}\left({\left[{IP}_{3}\right]}_{1}+{\left[{IP}_{3}\right]}_{2}+\ldots +{\left[{IP}_{3}\right]}_{n}-n\left[{IP}_{3}\right]\right),$$ (23)

where ${\left[{Ca}^{2+}\right]}_{i,1}$ … ${\left[{Ca}^{2+}\right]}_{i,n}$ are intracellular Ca^2+^ concentration, ${\left[{IP}_{3}\right]}_{1}$ … ${\left[{IP}_{3}\right]}_{n}$ are concentration in the connecting compartments, respectively.

The values of the diffusion rates from adjacent compartments to accepted compartment $D_{Ca}$ and $D_{{IP}_{3}}$ , depend on the compartment geometry and the inward and outward fluxes are different at the process branching sites (Gordleeva et al., 2019):

$$D_{Ca}=\frac{d_{Ca}S_{c}}{V_{i}d}$$ (24)

$$D_{{IP}_{3}}=\frac{d_{{IP}_{3}}S_{c}}{V_{i}d}$$ (25)

where $d_{Ca}$ and $d_{{IP}_{3}}$ is the diffusion constant for Ca^2+^ and IP_3_, respectively. $S_{c}$ is the cross-section area between compartments, $V_{i}$ is the volume of the compartment, d is the distance between the centers of compartments, for example, $d_{ns}$ in Figure 1B.

Table 1 gives the detailed description of parameters and their values used in the model.

**TABLE 1:** Model parameters.

Parameter	Value	Description	Source
N	20	Number of IP_3_Rs in a cluster	Shuai and Jung (2002)
$c_{0}$	2 μM	Free Ca^2+^	Ullah et al. (2006)
$c_{1}$	0.185	The ratio of ER volume to the cytoplasmic volume	Ullah et al. (2006)
$v_{1}$	6 s^-1^	Max Ca^2+^ channel flux	Ullah et al. (2006)
$v_{2}$	0.11 s^-1^	Ca^2+^ leak flux constant	Ullah et al. (2006)
$v_{3}$	2.2 μM/s	Maximum SERCA pump flux in primary process	Ullah et al. (2006)
$v_{5}$	0.025 μM/s	Rate of Ca^2+^ leak across the plasma membrane	Ullah et al. (2006)
$k_{1}$	0.5 s^-1^	Rate constant of Ca^2+^ extrusionc	Ullah et al. (2006)
$k_{3}$	0.05 μM	Dissociation constant of Ca^2+^ to SERCA in the parent process	Stamatakis and Mantzaris (2006)
	0.1 μM	Dissociation constant of Ca^2+^ to SERCA in nodes	Ullah et al. (2006)
$k_{4}$	1.1 μM	Dissociation constant for Ca^2+^ stimulation of IP_3_ production	Ullah et al. (2006)
$v_{4}$	2 μM/s	Maximal rate of IP_3_ production by PLCδ	Ullah et al. (2006)
$v_{\beta }$	0.062 μM/s	Maximal rate of IP_3_ production by PLCβ	Ullah et al. (2006)
$k_{g}$	0.78 μM	Dissociation constant for glutamate stimulation of IP_3_ production	Ullah et al. (2006)
g	1 μM	External glutamate	Ullah et al. (2006)
α	0.8	The relative effect of Ca^2+^ stimulation of PLCδ on IP_3_	Ullah et al. (2006)
$d_{1}$	0.13 μM	Dissociation constant for IP_3_	Ullah et al. (2006)
$d_{2}$	1.049 μM	Inactivation dissociation constant of Ca^2+^	Ullah et al. (2006)
$d_{3}$	0.9434 μM	Inactivation dissociation constant of IP_3_	Ullah et al. (2006)
$d_{5}$	0.08234 μM	Ca^2+^ activation constant	Ullah et al. (2006)
$a_{2}$	0.2 s^-1^	Ca^2+^ inhibition constant	Ullah et al. (2006)
$1/\tau_{ip3}$	0.14 s^-1^	Rate constant for loss of IP_3_	Ullah et al. (2006)
${\left[{IP}_{3}\right]}^{*}$	0.16 μM	Steady state concentration of IP_3_	Ullah et al. (2006)
$d_{ca}$	0.1 μm^2^/s	Diffusion constant of Ca^2+^	-
$d_{{IP}_{3}}$	1 μm^2^/s	Diffusion constant of IP_3_	-
$\alpha_{NMDA}$	2.2	Opening rate of NMDAR	Dehkordy et al. (2014)
$\beta_{NMDA}$	0.67	Closing rate of NMDAR	Dehkordy et al. (2014)
$V_{NMDA}$	0 mV	NMDA reversal potential	Destexhe et al. (1998)
$g_{NMDA}$	0.01–0.6 ns	NMDA conductance	Destexhe et al. (1998)
${\left[{Mg}^{2+}\right]}_{o}$	1–2 mM	External magnesium concentration	Destexhe et al. (1998)
η	0.012	Scaling factor controlling current carried by AMPAR	Dehkordy et al. (2014)
λ	0.12	Scaling factor controlling current carried by NMDAR	Dehkordy et al. (2014)

### 2.4 Tripartite synapse model

**FIGURE 2:**
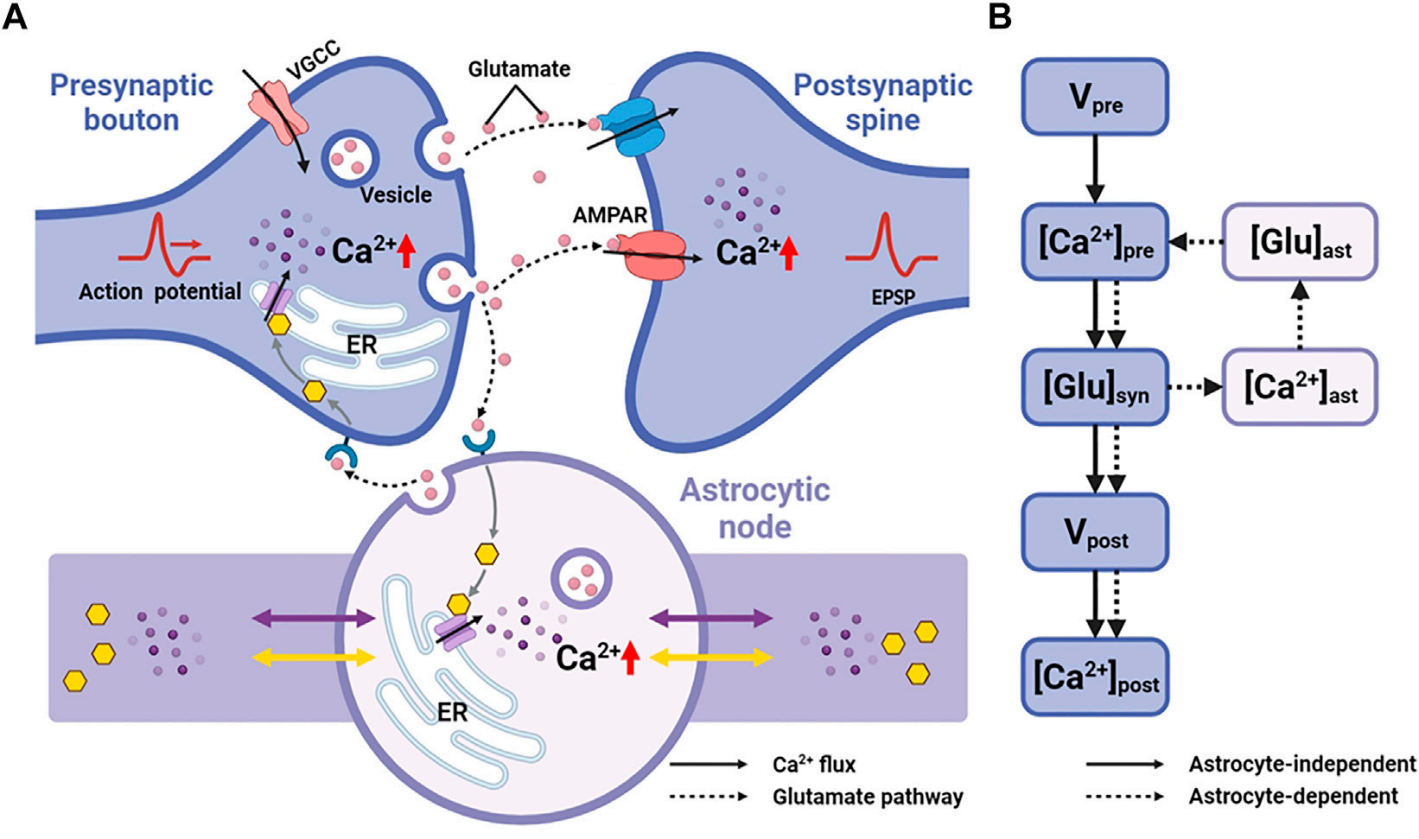
Information processing of tripartite synapse **(A)** Schematic of tripartite synapse consists of a presynaptic bouton, a postsynaptic spine and an astrocytic node. The action potential is generated at the presynaptic bouton and then elevates intracellular [Ca^2+^]. Increased [Ca^2+^] causes exocytosis of glutamate into the synaptic cleft. Synaptic glutamate leads to an increase in astrocytic [Ca^2+^]. Simultaneously synaptic glutamate binds with AMPAR and NMDAR causing an increase in postsynaptic membrane potential. Increased astrocytic [Ca^2+^] causes a release of glutamate. This part of glutamate then, in turn, feedbacks to the presynaptic neuron **(B)** Information flow of the tripartite synapse. The solid line shows the astrocyte-independent pathway, while the dashed line shows the astrocyte-dependent pathway.

Astrocytes can regulate synapses *via* Ca^2+^ signals in their fine processes of which nodes are thought to be the functional astrocytic component of synaptic structure (Arizono et al., 2020). Therefore, here, we replaced the astrocytic part of Tewari and Majumdar model (Tewari and Majumdar, 2012) with a node of node-shaft model and added some modifications. The main differences from the Tewari and Majumdar model are the Ca^2+^ kinetic part of the astrocytes and the addition of N-methyl D-aspartate receptors (NMDARs) to the postsynaptic neuron membrane. The brief descriptions of major neurophysiological steps are presented below, more details can be found in the (Tewari and Majumdar, 2012). Figure 2 showed the processes.

#### 2.4.1 Presynaptic action potential

The action potential of the presynaptic neuron due to the open and close of potassium, sodium and leak channels on the plasma membrane is given by (Tewari and Majumdar, 2012):

$$C\frac{dV_{pre}}{dt}=I_{app}-g_{K}n^{4}\left(V_{pre}-V_{K}\right)-g_{Na}m^{3}h\left(V_{pre}-V_{Na}\right)-g_{L}\left(V_{pre}-V_{L}\right)$$ (26)

where $I_{app}$ is the applied current density. m , n , and h represent activation and inactivation of the channels.

#### 2.4.2 Presynaptic Ca^2+^

The elevations of [Ca^2+^] in presynaptic bouton can be attributed to both action potential, denoted as ${\left[{Ca}^{2+}\right]}_{fast}$ and the intracellular release from ER, ${\left[{Ca}^{2+}\right]}_{slow}$ . Thus, the Ca^2+^ dynamics can be written as (Tewari and Majumdar, 2012):

$$\frac{d{\left[{Ca}^{2+}\right]}_{pre}}{dt}=\frac{d{\left[{Ca}^{2+}\right]}_{fast}}{dt}+\frac{d{\left[{Ca}^{2+}\right]}_{slow}}{dt},$$ (27)

where the rapid Ca^2+^ kinetics attribute to the construction (influx through voltage gated Ca^2+^ channels $J_{VGCC}$ and leakage $J_{PMleak}$ ) and destruction (efflux by Ca^2+^ pumps $J_{PMout}$ ) of Ca^2+^, respectively and are:

$$\frac{d{\left[{Ca}^{2+}\right]}_{fast}}{dt}=J_{VGCC}+J_{PMleak}+J_{PMout},$$ (28)

$$J_{VGCC}=-\frac{\overset{I_{Ca}}{\overbrace{\rho_{Ca}m_{Ca}^{2}g_{Ca}\left(V_{pre}-V_{Ca}\right)}}∙A_{bouton}}{z_{Ca}FV_{bouton}},$$ (29)

$$J_{PMleak}=v_{leak}\left({\left[{Ca}^{2+}\right]}_{o}-{\left[{Ca}^{2+}\right]}_{i}\right),$$ (30)

$$J_{PMout}=-\frac{\overset{I_{PMCa}}{\overbrace{v_{PMCa}\frac{{\left[{Ca}^{2+}\right]}_{i}^{2}}{{\left[{Ca}^{2+}\right]}_{i}^{2}+{k_{PMCa}}^{2}}}}∙A_{bouton}}{z_{Ca}FV_{bouton}}.$$ (31)

$I_{Ca}$ is the N-type channel current, $I_{PMCa}$ is the current due to electrogenic plasma-membrane Ca^2+^ ATPase, and $V_{Ca}$ is the Nernst potential of the channel. $A_{bouton}$ and $V_{bouton}$ are the surface area and volume of the bouton, respectively.

The form of ER-induced Ca^2+^ release is based on Li-Rinzel model with two additional equations governing ER [Ca^2+^] and [IP_3_]:

$$\frac{d{\left[{Ca}^{2+}\right]}_{slow}}{dt}=J_{chan}+J_{leak}-J_{SERCA},$$ (32)

$$\frac{d{\left[{Ca}^{2+}\right]}_{ER}}{dt}=-\frac{1}{c_{1}}\frac{d{\left[{Ca}^{2+}\right]}_{slow}}{dt},$$ (33)

$$\frac{d\left[{IP}_{3}\right]}{dt}=v_{g}\frac{g_{ast}^{0.3}}{g_{ast}^{0.3}+k_{g}^{0.3}}-\tau_{{IP}_{3}}\left(\left[{IP}_{3}\right]-{\left[{IP}_{3}\right]}^{*}\right),$$ (34)

where $g_{ast}$ is the glutamate released by astrocyte.

#### 2.4.3 Glutamate release from presynaptic neuron

The increased [Ca^2+^] and action potential train can lead to a transient increase in neurotransmitter release by vesicles. The kinetic model is (Tewari and Majumdar, 2012):

$$X\;\underset{\gamma }{\overset{5\theta C_{i}}{⇄}}\;X_{1}\;\underset{2\gamma }{\overset{4\theta C_{i}}{⇄}}\;X_{2}\;\underset{3\gamma }{\overset{3\theta C_{i}}{⇄}}\;X_{3}\;\underset{4\gamma }{\overset{2\theta C_{i}}{⇄}}\;X_{4}\;\underset{5\gamma }{\overset{\theta C_{i}}{⇄}}\;X_{5}\;\underset{\varepsilon }{\overset{\delta }{⇄}}\;X_{5}^{*},$$ (35)

where θ and γ are the Ca^2+^ association and dissociation rate constants respectively and δ and ε are Ca^2+^  independent isomerization constants. X represents the Ca^2+^ sensor, hence $X_{i}$ is the Ca^2+^ sensor with i Ca^2+^ bound, $X_{5}^{*}$ is the isomer of $X_{5}$ which is ready for glutamate release.

Additionally, glutamate can also be released as a spontaneous behaviour modeled by a Poisson process with the following rate when the presynaptic membrane is not depolarized (Tewari and Majumdar, 2012):

$$\lambda \left(C_{i}\right)=a_{3}{\left(1+\exp\left(\frac{a_{1}-C_{i}}{a_{2}^{*}}\right)\right)}^{-1}.$$ (36)

The fraction of releasable vesicles in the presynaptic neuron, effective vesicles in the synaptic cleft and inactive vesicles undergoing recycling are described by terms R , E , and I (Tewari and Majumdar, 2012):

$$\frac{dR}{dt}=\frac{I}{\tau_{rec}}-f_{r}∙R,$$ (37)

$$\frac{dE}{dt}=-\frac{E}{\tau_{inact}}+f_{r}∙R,$$ (38)

I=1−R−E, (39)

where $f_{r}$ is determined by a stochastic process corresponding to the number of vesicles ready to be released (0 or 1 or 2).

#### 2.4.4 Synaptic glutamate

The estimated glutamate concentration in the synaptic cleft can be represented mathematically as a source of vesicles and a clearance by neuron or astrocyte uptake (Tewari and Majumdar, 2012):

$$\frac{dg}{dt}=n_{v}∙g_{v}∙E-r∙g,$$ (40)

#### 2.4.5 Astrocyte node Ca^2+^

Glutamate can stimulus astrocytes *via* metabotropic glutamate receptors causing an increase of Ca^2+^ in astrocytic nodes. The dynamics are described in the first part of the modelling.

#### 2.4.6 Glutamate release from astrocyte

Astrocytes can release gliotransmitters in a Ca^2+^-dependent manner. Tewari and Majumdar assumed that only three Ca^2+^  ions bind with three independent gates or sites ( $S_{j},j=1,2,3$ ) can gliotransmitter release (Tewari and Majumdar, 2012):

$${Ca}^{2+}+C_{j}\underset{k_{j}^{-}}{\overset{k_{j}^{+}}{⇄}}O_{j},j=1,2,3$$ (41)

$$\frac{dO_{j}}{dt}=k_{j}^{+}∙{\left[{Ca}^{2+}\right]}_{ast}-\left(k_{j}^{+}∙{\left[{Ca}^{2+}\right]}_{ast}+k_{j}^{-}\right)∙O_{j},$$ (42)

where $C_{j}$ and $O_{j}$ are the closed and open rate of the gate, respectively. $k_{j}^{+}$ and $k_{j}^{-}$ are the opening and closing rates of the gate, respectively.

Another requirement of gliotransmitter release is that intracellular Ca^2+^ concentration should be higher than the threshold. Hence, the $R_{ast}$ , $E_{ast}$ , and $I_{ast}$ are modified as follows (Tewari and Majumdar, 2012):

$$\frac{dR_{ast}}{dt}=\frac{I_{ast}}{\tau_{rec}^{ast}}-\Theta \left({\left[{Ca}^{2+}\right]}_{ast}-{\left[{Ca}^{2+}\right]}_{ast}^{th}\right)∙\overset{O_{1}∙O_{2}∙O_{3}}{\overbrace{f_{r}^{ast}}}∙R_{ast},$$ (43)

$$\frac{dE_{ast}}{dt}=-\frac{E_{ast}}{\tau_{inact}^{ast}}+\Theta \left({\left[{Ca}^{2+}\right]}_{ast}-{\left[{Ca}^{2+}\right]}_{ast}^{th}\right)∙f_{r}^{ast}∙R_{ast},$$ (44)

$$I_{ast}=1-R_{ast}-E_{ast},$$ (45)

where Θ is the Heaviside function.

#### 2.4.7 Extra-synaptic glutamate

The released glutamate has the same form as Eq 39 (Tewari and Majumdar, 2012):

$$\frac{dg_{ast}}{dt}=n_{v}^{ast}∙g_{v}^{ast}∙E_{ast}-r_{ast}∙g_{ast},$$ (46)

#### 2.4.8 Excitatory postsynaptic potential

On the postsynaptic neuron membrane, NMDARs and α-amino-3-hydroxy-5-methyl-4-isoxazolepropionic acid receptors (AMPARs) are co-localized (Semyanov and Verkhratsky, 2021). Both of them can combine with the glutamate and then mediate the majority of excitatory neurotransmission and synaptic plasticity (Nakanishi, 1992; Traynelis et al., 2010). Consequently, we accounted for both AMPAR and NMDAR:

$$\tau_{post}\frac{dV_{post}}{dt}=-\left(V_{post}-V_{post}^{rest}\right)-\frac{r_{m}}{A_{spine}}∙\left(I_{AMPA}+I_{NMDA}\right),$$ (47)

$I_{AMPA}$ and $I_{NMDA}$ are AMPAR and NMDAR current, respectively. They are given by (Destexhe et al., 1998; Tewari and Majumdar, 2012):

$$I_{AMPA}=g_{AMPA}m_{AMPA}\left(V_{post}-V_{AMPA}\right),$$ (48)

$$I_{NMDA}=g_{NMDA}m_{AMPA}Mg\left(V\right)\left(V_{post}-V_{NMDA}\right),$$ (49)

where the gating variables $m_{AMPA}$ and $m_{NMDA}$ are associated with the synaptic glutamate concentration:

$$\frac{dm_{y}}{dt}=\alpha_{y}g\left(1-m_{y}\right)-\beta_{y}m_{y},y=AMPA,NMDA$$ (50)

$Mg\left(V\right)$ represents the magnesium block and can be model as (Destexhe et al., 1998):

$$Mg\left(V\right)=\frac{1}{1+\exp\left(-0.062V_{post}\right)∙{\left[{Mg}^{2+}\right]}_{o}/3.57}$$ (51)

#### 2.4.9 Postsynaptic Ca^2+^

Due to fractional Ca^2+^ current carried by AMPARs, NMDARs, Ca^2+^ elevations happen in the postsynaptic neuron. We simply modeled this process:

$$\frac{dc_{post}}{dt}=-\left(\left(\frac{\eta I_{AMPA}+\sigma I_{NMDA}}{z_{Ca}FV_{spine}}\right)+k_{s}\left(C_{post}-C_{post}^{rest}\right)\right)/\left(1+\theta \right),$$ (52)

All parameter values of the tripartite synapse model can be found in (Tewari and Majumdar, 2012) and Table 1.

### 2.5 Peak detection and analysis

The Ca^2+^ peaks were detected when ${\left[{Ca}^{2+}\right]}_{i}$ was higher than the following threshold: ${\left[{Ca}^{2+}\right]}_{mean}+n\sigma_{{Ca}^{2+}}$ , where ${\left[{Ca}^{2+}\right]}_{mean}$ was the mean Ca^2+^ concentration, $\sigma_{{Ca}^{2+}}$ was the standard deviation of ${\left[{Ca}^{2+}\right]}_{i}$ . n was set by hand depending on the signals, in this study, we chose n=2 to eliminate interference of the small Ca^2+^ fluctuations. The two indicators we used to characterize Ca^2+^ signals were the total number of peaks and mean amplitude of these peaks, respectively.

### 2.6 Simulation and code accessibility

In this paper, all simulations were performed with MATLAB (R2020a, MathWorks) and the code is available in supplementary materials. For each simulation experiment, we executed 20 trials with different random seeds depending on the system clock. So, the results presented in the figure were expressed as the mean ± STD of 20 simulations.

## 3 Results

**FIGURE 3:**
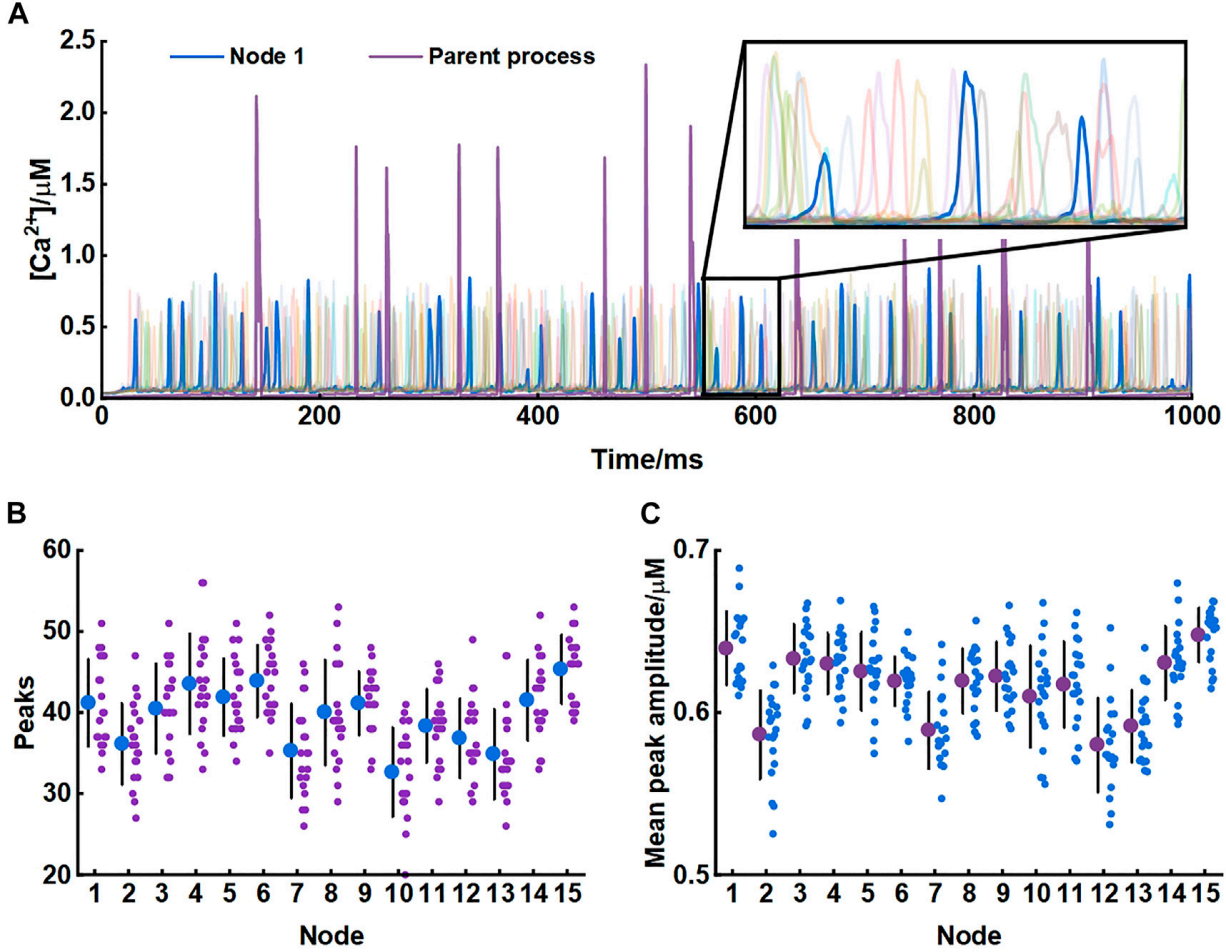
Astrocytic nodes exhibit microdomain Ca^2+^ signals **(A)** Ca^2+^ signals of all 15 nodes and the parent process. Blue line: Ca^2+^ signals of node 1; Purple line: Ca^2+^ signals of the parent process **(B)** The number of peaks of each node **(C)** The mean peak amplitude of each node.

Our model produced consistent Ca^2+^ signals essentially in agreement with results of experiments in live tissue (Arizono et al., 2020). Each node exhibited unique pattern of Ca^2+^ signal which were fast and small. While in the parent astrocytic process that nodes connected to, Ca^2+^ transients were larger both in amplitude and interval indicating significant differences in Ca^2+^ signals between the microdomain and larger astrocytic processes (Figure 3).

### 3.1 Scale size of astrocytic fine processes affect Ca^2+^ activity

We investigated the role of morphology of astrocytic fine processes on local Ca^2+^ activity with three morphological properties: width of nodes, width of shafts and length of shafts. All parameter values were based on the experimental data from Arizono et al. (2020). Nodes width, according to the experimental measurements, can range from 200 nm to 800 nm (median width: 330 nm) (Arizono et al., 2020). We therefore set the variations of width of nodes in our model from 0.2 μm to 0.8 μm. Simulations suggested that the number of Ca^2+^ peaks got more and the mean amplitude of peaks got higher with the increasing size of nodes, indicating larger nodes seemed to be more likely to generate Ca^2+^ events with high amplitude. Contrastly, only a few Ca^2+^ peaks were produced when nodes were tiny. The number of peaks and mean amplitude plateaued over a continuous width increase.

Hyperthin shafts are frequently below the diffraction limit of conventional light microscopy. Super-resolution microscopy shows they have a width from around 160 nm to 400 nm (median width: 202 nm) (Arizono et al., 2020). The size primarily distributes around 200 nm and only a very few shafts can reach a width close to 400 nm. So, in this study, we assumed that the width of shafts could vary from 0.1 μm to 0.4 μm. Results showed that both number and mean amplitude of peaks displayed a downward trend with increasing shaft width indicating that thin shafts were beneficial for nodes to generate larger signals.

Shaft length is the distance between two neighbor nodes and ranges from 0.4 μm to 2.4 μm (median length: 1.1 μm) (Arizono et al., 2020). In our study, this parameter was set from 1 μm to 4 μm. We found that the length of shafts hardly affected the local Ca^2+^ activity. Besides, all changes in morphology of fine processes did not affect Ca^2+^ signals of the parent process they connected.

**FIGURE 4:**
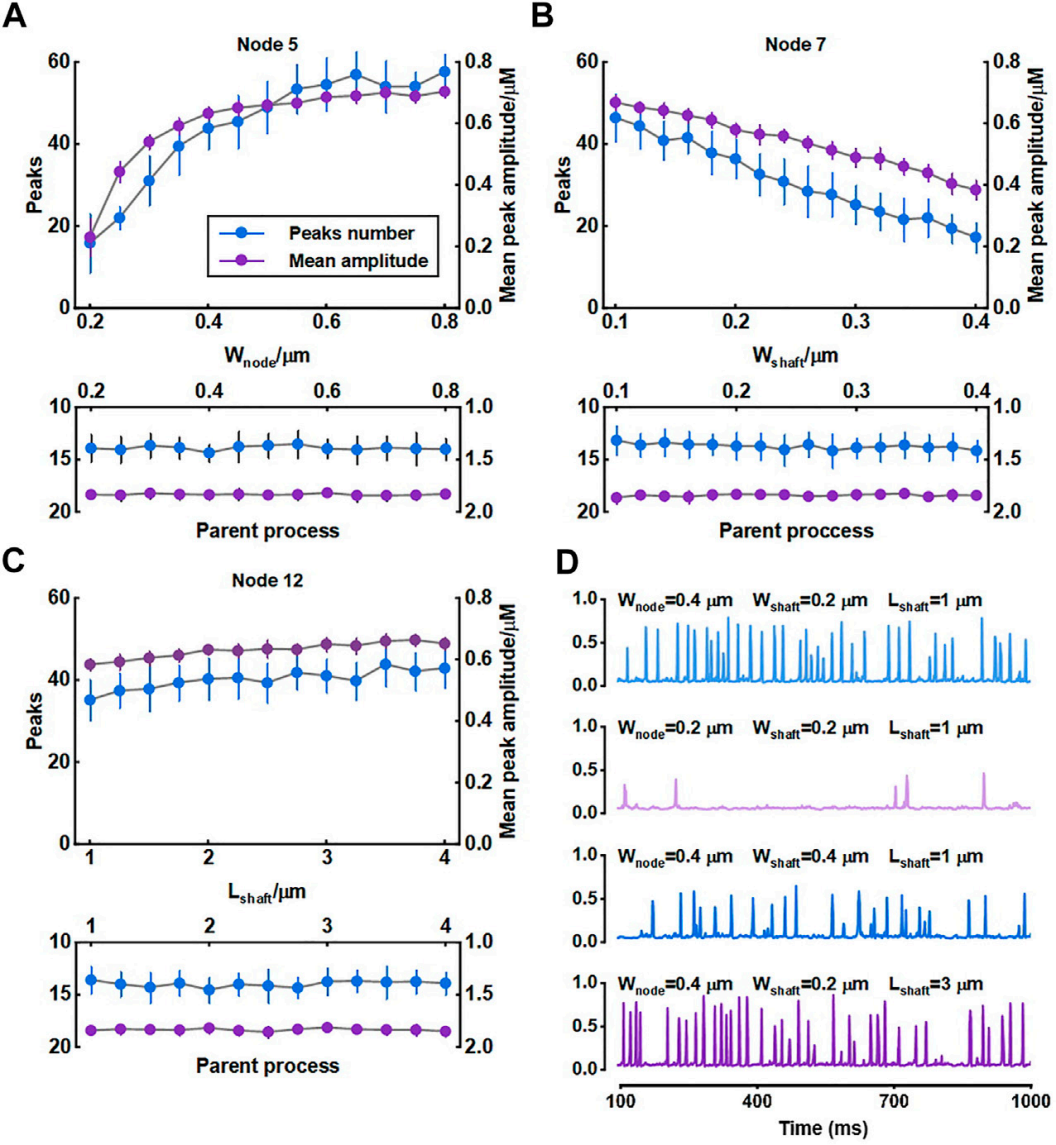
Effect of morphology of fine process on Ca^2+^ activity of fine and large processes **(A–C)** Quantification of the effect of node width, shaft width and shaft length on local Ca^2+^ signals of node 5, 7, and 12 and Ca^2+^ signals of the parent process. Data are represented as mean ± STD, n = 20 **(D)** Representative traces of local Ca^2+^ signals of node 5 with different morphology of fine processes.

For illustration, Figure 4D depictes the Ca^2+^ traces for four example parameter settings. The median values of width of nodes (330 nm), shafts (202 nm) and length of shafts (1.1 μm) measured in the experiments were reset as the control group ( $W_{node}=0.4\;\mu m$ ; $W_{shaft}=0.2\;\mu m$ ; $L_{shaft}=1\;\mu m$ ). As already mentioned, when nodes were minimal, Ca^2+^ peaks were rare and their amplitude was also low. Increasing the width of shafts decreased both the number of Ca^2+^ peaks and mean amplitude. Conversely, the longer shafts did not markedly influence the Ca^2+^ activity.

**FIGURE 5:**
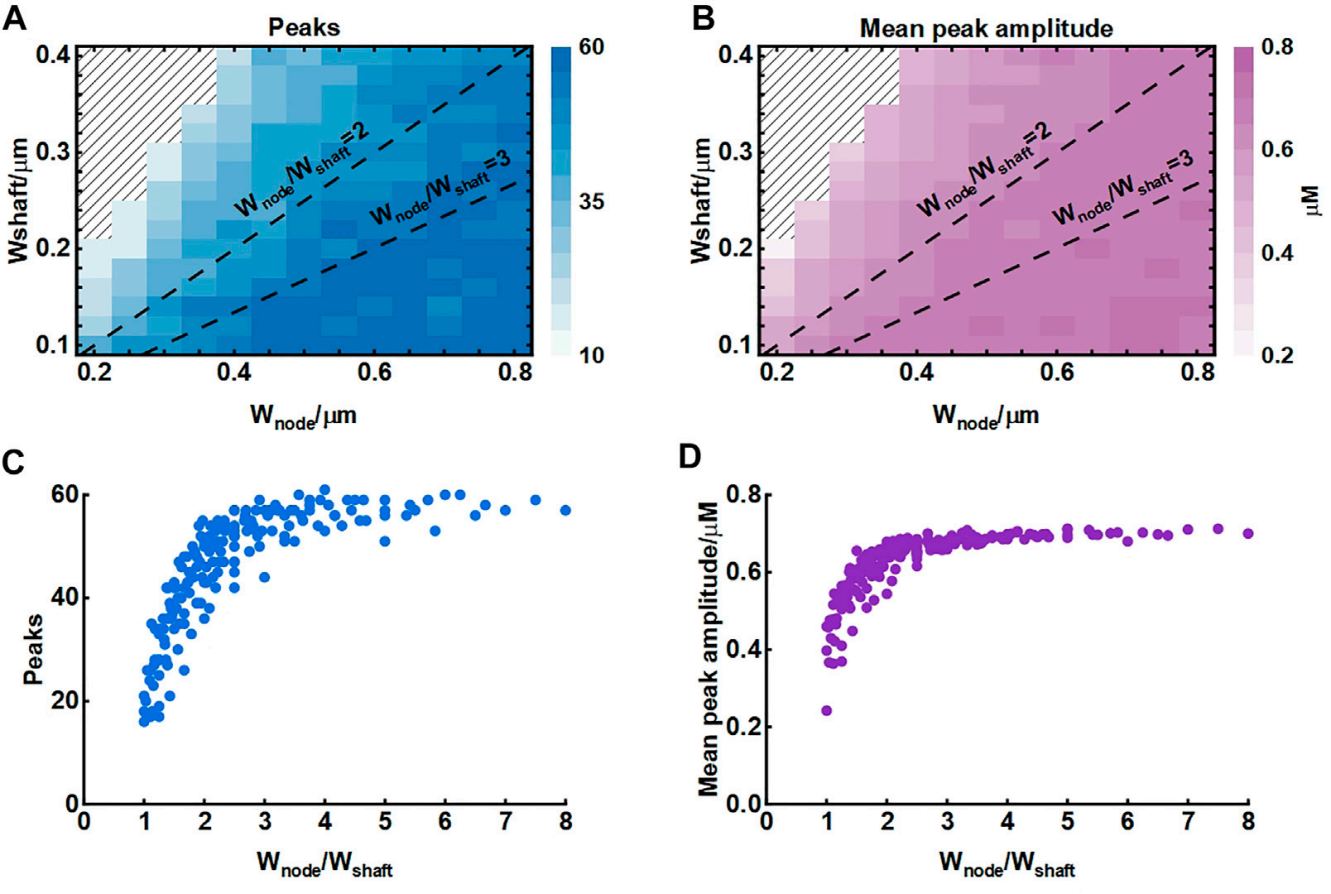
Effect of node and shaft width on local Ca^2+^ activity **(A)** Dependence of the number of peaks on node and shaft width. The shaded area represents. The dotted lines represent the width ratio of nodes to shafts is 2 and 3, respectively. Data are represented as mean, n = 20 **(B)** Dependence of the mean peak amplitude on node and shaft width **(C)** Dependence of the number of peaks on width ratio of nodes to shafts. Data are represented as mean, n = 20 **(D)** Dependence of the mean peak amplitude on width ratio of nodes to shafts.

The width of nodes and shafts strongly affected the shape of local Ca^2+^ activity (Figure 4) but what was responsible for the Ca^2+^ signal characteristic was unclear. To further explore the possible mechanisms, we simulated their co-effects on microdomain Ca^2+^ activity (Figure 5). Simulations suggested that the width ratio of nodes to shafts determined the local Ca^2+^ activity. Initially, both the number of peaks and mean amplitude showed an upward trend with the increasing ratio. When the ratio grew to about 3, the number of Ca^2+^ peaks was the most and the amplitude was also the highest. Subsequently, the continued increase of the ratio did not enhance the Ca^2+^ activity anymore. According to the frequency distribution of node and shaft width (Arizono et al., 2020), our results were likely to explain the physiologically potential relationships between nanostructure and functions such as diffusion requirements and information exchange, etc.

### 3.2 Connectivity of nodes to larger process affects astrocyte Ca^2+^ signaling

**FIGURE 6:**
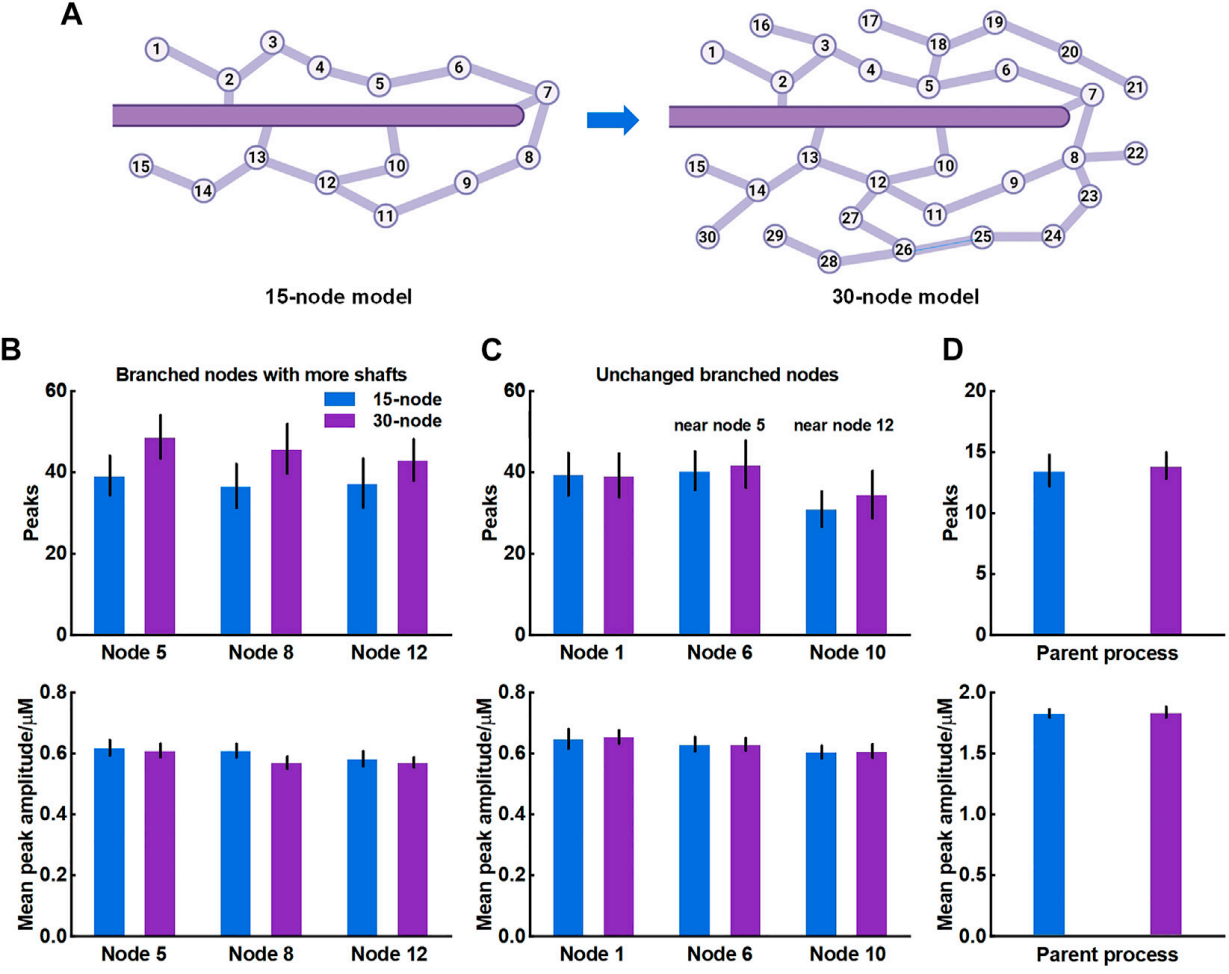
Effects of structure of microdomains on Ca^2+^ activity of fine and larger processes **(A)** 30-node model with more nodes and shafts **(B)** Quantification of the effect of different microdomain on local Ca^2+^ activity of changed branched nodes. Node 5, 8, and 12 are all branched points with more connecting shafts in 30-node model compared to 15-node model. Data are represented as mean ± STD, n = 20 **(C)** Quantification of the effect of different microdomain on local Ca^2+^ activity of unchanged branched nodes. Node 1, 6, and 10 are nodes with same connecting shafts both in the 15-node and 30-node model, where node 6 and 10 are neighboring nodes of node 5 and 12, respectively **(D)** Quantification of the effect of different microdomain on Ca^2+^ activity of the large process.

The crosstalk of Ca^2+^ signals between astrocytic primary and fine processes is unanswered. From the simulation results, Ca^2+^ activity of large process was not regulated by the nano-morphology (Figure 4). We speculated this might be linked to the structure of meshwork. To validate, we firstly constructed a 30-node model by adding more nodes and shafts to the original 15-node model (Figure 6A). We found that those nodes with more connecting shafts in the 30-node model (Node 5, 8 and 12) could produce more Ca^2+^ peaks and enhance their neighbor nodes (Node 6 and 10). This might be due to the increased diffusion fluxed from additional compartments. Although the mean amplitude of these peaks did not rise, the Ca^2+^ activity seemed to be more active. While the distal node (Node 1) which was away from the changed branched nodes barely changed. Figure 6D indicated that the Ca^2+^ activity of large process was either not affected by the structure of the microdomain.

**FIGURE 7:**
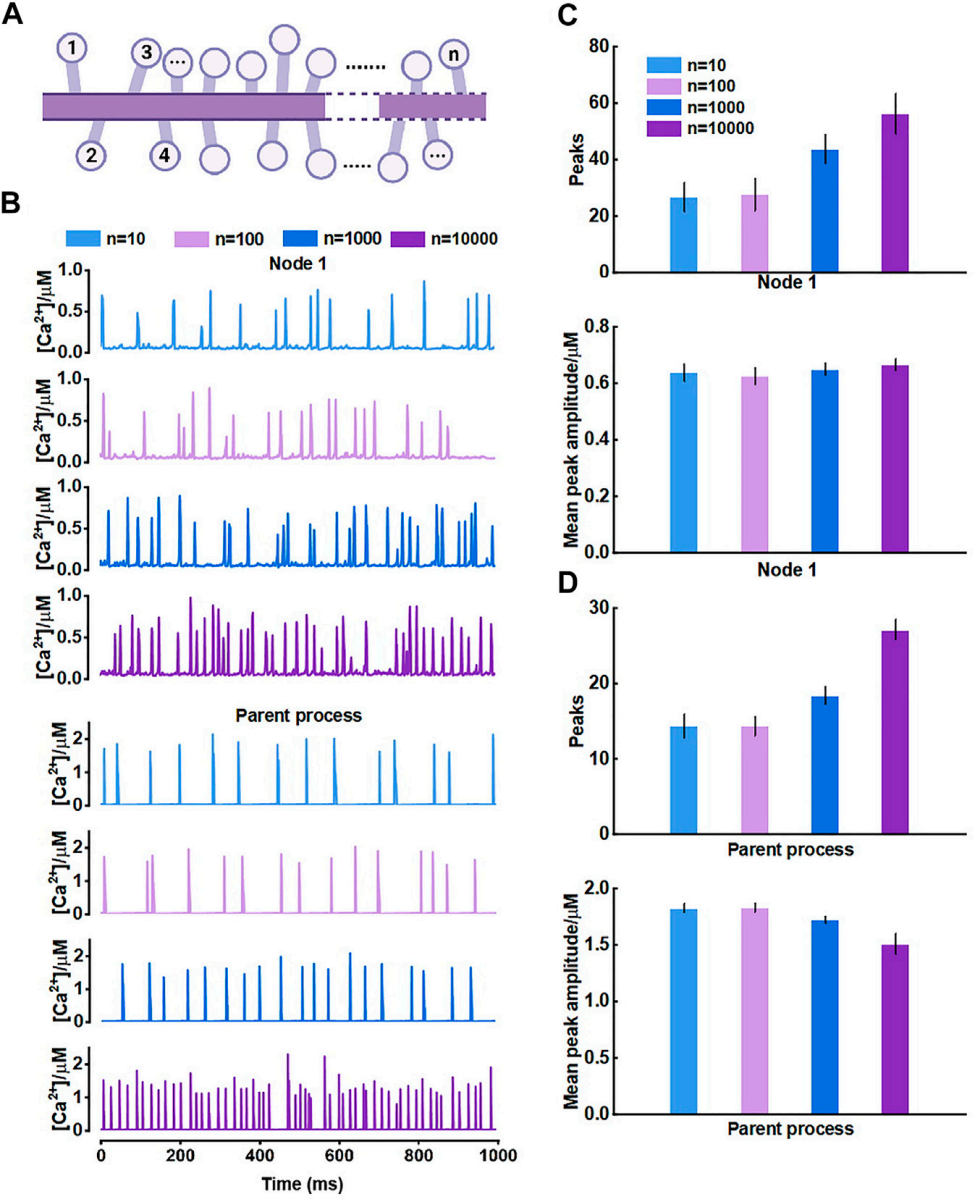
Effects of the number of nodes connected to the large process on Ca^2+^ activity **(A)** Schematic representation of the model which only contains nodes that are connected to the parent process **(B)** Representative traces of Ca^2+^ signals of node 1 and the parent process with different number of nodes, respectively **(C)** Quantification of the effect of different number of nodes on local Ca^2+^ activity **(D)** Quantification of the effect of different number of nodes on Ca^2+^ activity of large process.

For the results, we thought it should be the morphology-determined different Ca^2+^ exchange between compartments altering the mutual Ca^2+^ activity but seemed to need cumulative effects, i.e., enough compartments and fluxes. Hence, we then proposed another model consisting of only those nodes connected to the parent process (Figure 7A) and investigated how they interrelated. The results of model simulations corroborated the rationality of our hypothesis. Figure 7B shows that the Ca^2+^ activity of nodes was enhanced with the increasing number of nodes. In turn, the Ca^2+^ signals of the large process were affected simultaneously. The frequency of peaks got higher but the peak amplitude decreased. We figured the potential reason was the imbalance of the diffusion between two different-size compartments.

Overall, this part revealed the crosstalk between astrocytic fine and larger processes. The number of nodes in a subdomain and the connectivity of nodes to larger processes strongly affected the Ca^2+^ activity of each compartment. This effect only emerged when there were sufficient numbers of nodes connected to the larger process.

### 3.3 Morphological deficits influence the synaptic transmission

The fine structure of astrocytes is highly plastic and activity-dependent, which grant astrocytes the ability to adapt to various environments and regulate synapses, blood vessels and other cells. Particularly in many pathological conditions, the morphological changes are vital (Procko et al., 2011; Bellesi et al., 2017; Schiweck et al., 2018; Zhou et al., 2019). However, the underlying mechanisms and the significance of the astrocyte structural alterations are not yet clear. Here, a tripartite synapse model based on the node-shaft model was constructed to explore the potential impact (Figure 2).

In the previous simulations, we especially noticed that when nodes are small, Ca^2+^ activity in this condition is exceptionally inactive compared to other situations (see Figure 4A; $W_{node}=0.2\mu m$ ). Actually, in many neurological diseases, astrocytes will display a type of morphological deficit, atrophy, defined as decrease in surface area and volume of morphological profiles, particularly manifested in diminution of fine processes (Verkhratsky et al., 2019). Therefore, we associated small nodes with atrophy and assumed two possible pathways that were possibly involved in pathology as below.

**FIGURE 8:**
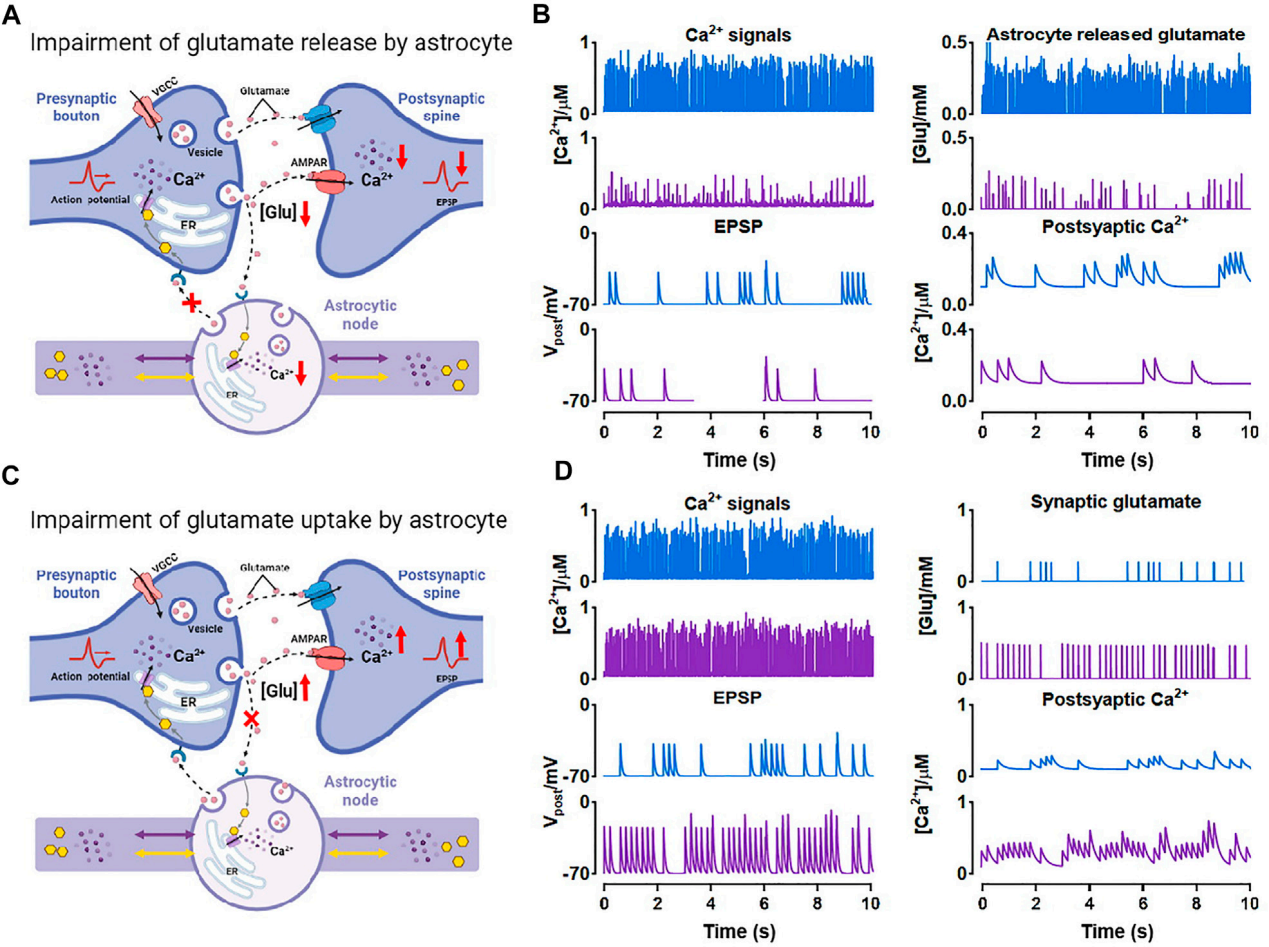
Effects of morphological deficits of nodes on synaptic transmission **(A)** Reduced node impairs Ca^2+^-dependent glutamate release **(B)** Comparison between the normal size (blue) and reduced size (purple) of node on synaptic signals **(C)** Reduced node impairs glutamate uptake by astrocyte **(D)** Comparison between the normal size (blue) and reduced size (purple) of node on synaptic signals.

As shown, both the number of Ca^2+^ peaks and their amplitude were low in small nodes. Simultaneously, the release of glutamate is Ca^2+^-dependent manner (Tewari and Majumdar, 2012), therefore, this low Ca^2+^ level can strongly affect the glutamate release of the astrocyte. Figure 8A showed the process. When atrophy or deficits happened, Ca^2+^ concentration hardly reached the release threshold, hence, there was only a little glutamate released to the synaptic cleft causing the simultaneous reduction of excitatory postsynaptic potential (EPSP) and postsynaptic Ca^2+^ dynamics. Glutamate is the predominant excitatory neurotransmitter, hence the abnormally low level of glutamate probably leads to a decrease in brain excitability.

Also, glutamate can be a potential excitotoxin. We considered another possible situation which is shown in Figure 8C. When the node size decreases, the astrocytic coverage of synapse may diminish and the ability of glutamate reuptake by astrocyte can be therefore weakened (Verkhratsky et al., 2019). We simulated the accumulation and increase of glutamate in synaptic cleft by raising the probability of glutamate release by presynaptic neuron and downregulating the glutamate clearance rate simultaneously. Then the accumulated glutamate in the synaptic cleft elevated both postsynaptic Ca^2+^ and EPSP, eventually leading to NMDAR-mediated excitotoxicity.

In general, our simulations indicated that astrocyte structural changes could impair the glutamate-dependent synaptic transmission and affect the downstream processes closely connected to memory, cognition, and other advanced brain functions (Schiweck et al., 2018; Zhou et al., 2019).

## 4 Discussion

Astrocytes use “Ca^2+^ language” to process information and most of the basal Ca^2+^ activity occurs in their fine structures where they compose the spongiform domain (Bindocci et al., 2017). This attractive area has gained great attention but also faced challenges and difficulties from techniques. Deciphering the mechanistic link between nano-morphology and intracellular Ca^2+^ signals is essential yet hard to analyze experimentally. In this study, we studied the role of fine morphology of astrocytes by using computational tools and data from super-resolution microscopy. Our model validated the effect of morphology of astrocytic fine processes on local Ca^2+^ activity and further showed that the width ratio between nodes and shafts determined the shape of Ca^2+^ signals in microdomain. How the inextricably related players, fine and large processes, interact remains unclear. Here we provided a computational study indicating that the differences between compartmental diffusion might be an explanation and the number of fine processes connected to the large processes could affect the mutual Ca^2+^ activity. Since astrocytic fine processes actively participate in the synaptic communication forming the so-called tripartite synapse, we associated the node-shaft model with the existing tripartite synapse model. No doubt, this could be an essential model of astrocyte-neuron networks. The results showed the strong influence of reduced morphology of nodes on synaptic transmission.

Actually, Denizot et al. have proposed a model with realistic 3D geometries of fine processes after their super-resolution investigation (Arizono et al., 2020; Denizot et al., 2022). They designed an isolated section of fine process with five identical nodes connected by four identical shafts. Nodes are approximated as spheres of width $d_{0}$ = 0.38 μm and shafts as 1 μm-long cylinders. The width of shafts has three different values: $d_{shaft}=d_{0}$ , $d_{shaft}=d_{0}/2$ and $d_{shaft}=d_{0}/3$ . They focused on the effect of shaft width on Ca^2+^ signals. They found that thin shafts favor node compartmentalization and more robust signal propagation, simultaneously enhance Ca^2+^ activity in nodes. Contrastly, we here built a more comprehensive model of the spongiform structure and investigated the width of nodes and the length of shafts. Our model showed that large nodes were also beneficial for enhancing Ca^2+^ activity while shaft length had no effect. Besides, we studied the crosstalk between the fine and large processes. We also discussed the role of morphological changes in fine processes on synapses. Although Denizot et al. mentioned the impact of astrocytic nano-morphology on astrocyte activity at tripartite synapses in health and disease, it seems to lack more detailed analysis and validation.

In addition, Savtchenko et al. and Denizot et al.‘s earlier studies also proposed astrocyte models associated with the nano-morphology of astrocytic fine processes (Savtchenko et al., 2018; Denizot et al., 2019). Savtchenko et al. transformed polygonal z-stacks representing 3D-reconstructed processes from electron microscopy into z-stacks of cylindrical slabs *in silico*. They only briefly compared two modelled astrocytes with different stem trees that were populated with the same nanoscopic processes. Collectively, the contribution of this work is more for a powerful tool provided to study functions of astrocytes than astrocytic physiology itself. Denizot et al. mimicked an astrocytic process consisting of a cylinder of length 1 μm and radius 0.1 μm. They investigated some mechanisms on astrocyte Ca^2+^ microdomains including clustering of IP_3_R channels, endogenous buffers, etc. The two studies are helpful but they both only regard the processes as simple cylindrical structures, also do not allow for that nodes are preferential sites of Ca^2+^ initialization and synaptic transmission. One necessary task of computational biology is to incorporate the latest experimental data into our modeling work to advance the construction and validation of models.

Astrocytic fine processes possess a very high surface-to-volume ratio, and this parameter determines the probability of Ca^2+^ events initiation (Wu et al., 2019) and is necessary for efficient glutamate uptake (Patrushev et al., 2013). In our simulations, we found a specific width ratio of nodes to shafts, about 3, was the most favorable structural ratio under which the local Ca^2+^ activity was the most active. We thought this ratio presented in astrocytes might be advantageous to diffusion and explained why Ca^2+^ events more frequently start in thin astrocytic processes (with a higher ratio) than in thick processes or Soma (Semyanov, 2019). Especially, when astrocytes exhibit morphological deficits or undergo reactive response in diseases, their volumes and structures change, which is sure to induce the alterations in Ca^2+^ activity, eventually leads to behavioral modification of astrocytes like the loss of functions or enhanced protection.

Astrocytic Ca^2+^ activity seems to be compartmentalized. Experiments show that most of Ca^2+^ signals that reach the interface between two structures (like Soma and processes) stop there just like some forms of physical and/or biological barrier are presented at the interfaces (Bindocci et al., 2017; Arizono et al., 2020). How the other small portion of events crosses the barrier and propagates from one to another compartments, such as processes to Soma, remains unanswered, which still needs more experimental explorations. Therefore, in this model, the assumed compartments were all non-confined. We mainly focused on the crosstalk between the fine and large processes and found that the connectivity of nodes to larger processes markedly shaped the Ca^2+^ signal of the large structures. This finding may make sense because in many neurodegenerative instances, the change in number of astrocytic processes is a frequent occurrence, for example, reactive astrocytes always have more complex branchy degree while atrophic astrocytes have less processes because of morphological deficits (Zhou et al., 2019).

Astrocytes are important shapeshifters which allow them to respond to the changing environment during development, injury and disease. Their morphological changes have been observed in various experiments especially in pathological conditions, but most mechanisms remain unknown. For example, hypertrophic reactive astrocytes are always thought to be a hallmark in many neurodegenerative diseases, while the atrophic astrocytes are presented earlier in AD (Verkhratsky et al., 2016; Jones et al., 2017; Escartin et al., 2021). So far, the molecular basis for astrocyte atrophy preceding astrogliosis, and what is the contribution of this phenotype to the onset of AD are still puzzles. Moreover, the apparent morphological deficits including reduced volumes of fine processes are also observed in Huntington’s disease (Octeau et al., 2018). Instead, in Parkinson’s disease, there is a significant expansion of the coverage by astrocyte processes of striatal tripartite synapse with unknown of the underlying mechanisms (Villalba and Smith, 2011). According to our simulations, the atrophy of astrocytic fine processes altered astrocytic Ca^2+^ activity thereby affecting the release of glutamate. The degradation of the excitability was hence the possible reason for early cognitive disorder and memory loss in the early stage of AD. This conclusion is an interesting idea but we realize and believe that more wet-lab design and data are required to address this specific clinical question. On the other hand, our model explained that the increase of the astrocytic coverage could enhance glutamate uptake in the synaptic cleft and decrease the high extracellular glutamate levels and overall neuronal hyperactivity which was consistent with the experimental results in the parkinsonian state (Villalba and Smith, 2011). Overall, the agreement between our model and experiments potentially illustrates that astrocyte-induced synaptic impairment may be responsible for the dysfunction of advanced brain abilities such as cognition, memory, etc. Rescuing the morphological deficits and guarantying the morphological integrality are potentially the cure for neurological disorders.

Knowledge about Ca^2+^ microdomain is rapidly increasing. A recent study indicates that ER is observed in only ∼45% of fine processes (Aboufares El Alaoui et al., 2020) and other sources of these dynamics are therefore revealed. Clear evidence has shown that extracellular Ca^2+^ influx through cell membrane ion channels can contribute to astrocytic microdomain events with fast dynamics, such as TRPA1 and Na^+^/Ca^2+^ exchanger (Ahmadpour et al., 2021). Besides, Agarwal et al. (Agarwal et al., 2017) found that in the absence of IP_3_-dependent release, microdomain Ca^2+^ transients still occur due to the opening of the mitochondrial permeability transition pore. These various fluxes can also induce cascade reaction. For instance, once store-operated Ca^2+^ entry is activated, the depletion of ER activates the ER Ca^2+^ sensors stromal interaction molecules 1 and 2, then the molecules translocate to the junctional ER to interact with and activate store-operated Ca^2+^ release-activated Ca^2+^ channels (Toth et al., 2019), suggesting that intracellular release and transmembrane influxes coordinately contribute to Ca^2+^ microdomains. Considering a complete picture of the Ca^2+^ microdomain would definitely help better understand the theme but also is challenging due to the missing explanation of some mechanisms. In this study, we aimed to provide a biologically plausible theoretical framework that could provide reasonable predictions on the morphology-defined Ca^2+^ microdomains that are difficultly enabled in experiments.

In fact, our model leaves much to be desired and needs further improvement. Firstly, our node-shaft model is only an approximation of the astrocyte microdomains and cannot fully reflect the morphology of fine processes. Secondly, our model does not fully consider the factors affecting astrocyte Ca^2+^ dynamics. In astrocytes, in addition to glutamate, ATP, GABA and other signaling pathways also affect Ca^2+^ signalization and contribute to the regulation of a number of both physiological and pathophysiological processes in the nervous system as well (Chen et al., 2023). The model only examined a subset of the astrocyte microdomains and did not take into account the extracellular Ca^2+^ diffusion. Thirdly, Some details of the model are not rigorous enough. The presence of ER in the fine processes is controversial, but there is evidence that Ca^2+^ microdomains can have a pure intracellular or extracellular Ca^2+^ source (Lia et al., 2021). And we chose to add ER in the thinnest astrocyte processes for some considerations, thus ignoring another possibility. What’s more, we did not consider ryanodine receptors in our modeling, which is something we need to explore in the future. The Hodgkin-Huxley model is used to generate presynaptic spikes without using a fluctuating applied current. Instead a constant current is used here because we found that even substituting 1, 2, and 5 times the noise of the 0–1 uniformly distributed current has little effect on the results. This may be partly due to the short current window we applied. Finally, astrocytes share their cytoplasm through gap junctional coupling into a syncytium. The node-shaft model can be extended to describe the communication between two astrocyte microdomains, and even to astrocyte networks. On the other hand, our tripartite synaptic model, as the smallest unit of signal propagation of astrocyte-neuron network, is also helpful for future network construction. By further understanding the astrocyte microdomain in the future, we hope to build more realistic astrocyte models with more comprehensive signaling pathways.

Although many questions and challenges exist, Ca^2+^ signals in astrocyte microdomains have gained a decade of significant advances, as said, a new decade of great discoveries has just begun (Lia et al., 2021). We need fruitful partnerships between tool generators, modellers and experimentalists to catalyze major breakthroughs in biology, physiology and pathology of astrocytes.

## Data Availability

The original contributions presented in the study are included in the article/supplementary material, further inquiries can be directed to the corresponding author.
